# Становление эндокринологии Древнего мира

**DOI:** 10.14341/probl12878

**Published:** 2022-02-04

**Authors:** А. Н. Куринова, Т. В. Никитина, Д. А. Трухина, Е. А. Пигарова, А. А. Трухин, В. С. Ясюченя

**Affiliations:** Национальный медицинский исследовательский центр эндокринологии,; Национальный медицинский исследовательский центр эндокринологии,; Национальный медицинский исследовательский центр эндокринологии,; Национальный медицинский исследовательский центр эндокринологии,; Национальный медицинский исследовательский центр эндокринологии,; Национальный медицинский исследовательский центр эндокринологии,

**Keywords:** эндокринология, Древний мир, зоб, щитовидная железа, сахарный диабет, гигантизм.

## Abstract

Формированию эндокринологии как самостоятельной дисциплины предшествовало многовековое накопление опыта врачевания. Медицина древнего мира развивалась на основе преемственности — в соответствии с основным принципом «relata refero» (рассказываю рассказанное). Несомненно, в странах Древнего мира представление о лечебном деле и фармации во многом было схоже, но в то же время каждая цивилизация имела свои географические, исторические и культурные особенности.

В Древнем мире к наиболее изученным эндокринным патологиям относилась патология щитовидной железы. Упоминание об эндемическом зобе встречается в трудах врачей Древнего Китая, Древней Индии и Древней Греции. И хотя взаимосвязь йода и щитовидной железы в то время была неизвестна, но увеличенную в объеме шею повсеместно лечили путем приема водорослей и высушенных морских губок.

Болезни эндокринной системы описаны во многих исторических источниках, так, в Библии описан гигантизм, в Талмуде — гиперпролактинемия.

Особое внимание уделялось изучению сахарного диабета, однако вплоть до XX века патогенез и лечение заболевания все еще оставались неизвестны.

## Abstract (en)

The formation of endocrinology as an independent medical discipline was preceded by the accumulation of medical experience during many centuries. The medicine of the ancient times was developing on the basis of continuity according to the basic principle «relata refero» (I tell what I have been told). Medicine and pharmacy in the countries of the ancient world had many similarities, but at the same time each civilization had its own geographical, cultural and historical particularities. The pathology of the thyroid was among the most studied pathologies in Ancient world. There are frequent mentions of the endemic goiter in the works of doctors from Ancient China, Ancient India and Ancient Greece.

Although the link between iodine and the thyroid was not known, algae and dried sea sponges were using for treating swollen neck.

There are many descriptions of neuroendocrine pathologies in historical sources, for example the Bible describes gigantism and the Talmud — hypoprolactinaemia.

Special attention was paid to the study of diabetes mellitus, although the pathogenesis and treatment of the disease remained unknown until the 20th century.

## ДРЕВНИЙ ВОСТОК

Становление медицины берет свое начало в Древнем Египте. Изучение памятников древнеегипетской культуры позволяет получить наглядное представление о жизни населения. Одной из самых распространенных эндокринных патологий, изображенных на древних картинах, является зоб. Рельеф Клеопатры с увеличенной в объеме шеей, увековеченный в храмовом комплексе в Дендере, свидетельствует о наличии у царицы патологии щитовидной железы. В скульптурах Древней цивилизации можно проследить увеличенные черты лица Эхнатона, евнухоидный тип телосложения, которые могли быть связаны с аденомой гипофиза и наличием у фараона акромегалии [1].

Первое упоминание о сахарном диабете отражено в знаменитом медицинском манускрипте Древнего Египта – Папирусе Эберса (около 1550 г. до н. э.). В нем описаны характерные признаки диабета, такие как похудение («тело сморщено, словно заколдованное»), кожные нарушения, жажда, и предположение о локализации источника болезни (у желудка, но не связанное с самим желудком) [2][3]. Однако египетский эндокринолог и историк медицины Пауль Гхаллиоунгуи отмечает, что в медицинском папирусе более раннего периода, Кахунском (около 1850 г. до н. э.), посвященном лечению женских болезней, приводится рецепт под названием: «Лечение женщин, испытывающих невыносимую жажду»*.

Древнеегипетских врачей можно считать предшественниками современных специалистов: диабетологов, нейроэндокринологов, тиреоидологов, что подтверждал Геродот: «Медицина практиковалась по принципу разделения: один врач лечил только одно заболевание» [1].

Древний Египет оказал огромное воздействие на становление медицины Древнего мира, в первую очередь государств Ближнего Востока.

Основными источниками знаний о древнееврейской медицине являются Библия и Талмуд. Согласно Ветхому Завету, здоровье — это дар Бога, а болезнь — проявление Его гнева, предотвратить или отвести который можно только подчинением, искуплением, молитвой или принесением жертвы.

В Библии содержится самое раннее литературное описание семейного гигантизма. Голиаф, знаменитый филистимлянский воин, был ростом «6 локтей с пядью» или 2,772 м; близкие родственники известного библейского персонажа (отец и 3 брата) также были гигантами. При акромегалии и гигантизме одним из основных симптомов является артропатия. Так, наколенники, о которых говорится при описании доспехов Голиафа, нужны были ему, вероятно, не столько для защиты, сколько для того, чтобы помогать слабым связкам ног поддерживать огромный вес тела. По всей видимости, необходимость в оруженосце, подсказывавшем Голиафу дорогу, свидетельствует о развитии у гиганта хиазмального синдрома вследствие компрессии перекреста зрительных нервов аденомой гипофиза. Хрупкость костей черепа, ставшая причиной гибели Голиафа от камня, выпущенного Давидом из пращи и попавшего в лоб, может быть обусловлена как повышенной резорбцией костной ткани при изолированной опухоли аденогипофиза, приведшей к гигантизму, так и гиперпаратиреозом в рамках синдрома множественных эндокринных неоплазий 1 типа [5].

По одной из версий в Ветхом Завете описан эпизод гипогликемии у Исава: голод был настолько невыносим, что Исав «продал свое право первородства за чечевичную похлебку брату Иакову»**. Однако утверждать, страдал ли Исав какой-либо эндокринной патологией, сопровождающейся наличием гипогликемического синдрома, не представляется возможным. Известно, что в отличие от своего брата-близнеца, у Исава был гипертрихоз (ивр.  ‏עֵשָׂו‏‎, «Эсав» — волосатый)***.

В Талмуде был впервые упомянут один из наиболее распространенных нейроэндокринных синдромов — гиперпролактинемия: мужчина, жена которого умерла во время родов, кормил грудью своего ребенка из-за отсутствия возможности нанять кормилицу**** [1].

Традиционная система индийской народной медицины, Аюрведа, зародилась в 1400 году до н. э. Ее основу составляли труды, передаваемые из поколения в поколение на протяжении тысячелетий.

Основоположник аюрведической медицины Ашрайя Чарака (ок. 300–200 год до н. э.) передал знания своего века о патологии щитовидной железы «Галаганда» (зоб) в трактате о внутренних болезнях «Чарака-самхита». Он классифицировал патологию на ватайя-галаганда (тиреотоксический), кафайя-галаганда (гипотиреоидный) и медайя-галаганда (кистозный). Чарака предлагал лечить гипотиреоз продуктами питания, например, употреблением риса, молока, ячменя, ограничивая при этом содержание кислых продуктов в рационе. Фитотерапия гипотиреоза заключалась в приеме баугинии (Bauhinia veriegata), бурых водорослей (Fucus vesiculosus), гуггула (экстракт, выделенный из смолы дерева Commiphora mukul), а также пурнавы (boerhaavia diffusa Linn), обладающей мочегонным свойством и, как следствие, уменьшающей отеки [7]. В случае наличия зоба, сопровождавшегося такими симптомами, как затрудненное дыхание, выраженная общая слабость, отсутствие аппетита и голоса, а также при длительности заболевания более года, патология считалась неизлечимой [1].

Больным с симптомами тиреотоксикоза рекомендовали употреблять растения рода Зюзник (Lycopus virginicus, Lycopus europaeus и др.) [7].

В Аюрведе фигурирует упоминание и о сахарном диабете — «мадхумеха» (madhu — мед, meha — моча). Древнеиндийские врачи заметили, что заболевание, проявлявшееся полиурией и полидипсией, часто наблюдалось у состоятельных людей, в рационе которых содержалось большое количество богатых углеводами продуктов [8].

В древнекитайских текстах, датируемых 2700 годом до н. э., также содержатся описания зоба. В 85 г. древнекитайский медик Цуй Чинди разделил опухоли шеи на солидные (злокачественные) и подвижные (доброкачественные) [6].

Во II веке знаменитый врач и фармаколог династии Хань, «китайский Гиппократ» Чжан Чжунцзин (150–219 гг.) изучал сахарный диабет, который рассматривался как«Сяо Хо» — «изнуряющая жажда» [1].

## ДРЕВНЯЯ ГРЕЦИЯ

«Отец медицины» Гиппократ (460–370 гг. до н. э.) жил в период рассвета классической греческой культуры. Знаменитый врач и философ является автором сочинений «Corpus Hippocraticum» (Сборник Гиппократа), посвященных медицине.

В своей работе «De Glandulis» («О железах») Гиппократ пишет следующее:

«… плоть желез отличается от остальной части тела, будучи губчатой и полной вен;

они находятся во влажных частях тела…

… и мозг является железой так же, как и молочные железы» [1].

Гиппократ считал щитовидную железу «увлажнителем» вдыхаемого воздуха, также предписывая данное свойство слюнным железам. По мнению целителя и его учеников, зоб является лишь эстетическим дефектом, возникающим из-за употребления талой воды [1][9].

Также в сочинении «De Glandulis» древнегреческий целитель отмечает: «…большинство заболеваний желез не являются первичными, и когда железы поражаются, то становятся бугристыми, образуя зоб, а тело охватывает лихорадка» [1][6][7].

В труде «О женских болезнях» упомянута связь ожирения и бесплодия.

## ГРЕКО-РИМСКИЙ ПЕРИОД

Наиболее полные сведения о римской медицине времен ранней Империи получены благодаря Авлу Корнелию Цельсу и Аретею из Каппадокии — выдающимся ученым-энциклопедистам.

Авл Корнелий Цельс (25 г. до н. э. — 50 г. н. э.), обладая практическими медицинскими знаниями, составил обширные трактаты в области медицины, в том числе состоящий из 8 книг трактат «О медицине» («De Medicina»), датируемый 30 г. Следует отметить, что в своей работе Цельс использовал и знания, полученные из греческих источников.

Книги включают в себя описания болезней, хирургические манипуляции, рецепты лекарств. Так, описан сахарный диабет (как заболевание, проявляющееся полиурией, слабостью). В качестве лечения прописывалась диета, главным аспектом корой являлось ограничение количества принимаемой пищи [1].

Цельс подробно описал опухоли шеи. В тринадцатой главе седьмой книги трактата повествуется об образованиях щитовидной железы: «На шее между кожей и трахеей возникает опухоль, называемая греками “бронхоцеле”, которая по структуре может быть либо полностью мясистой, либо содержать жидкость наподобие меда или воды, а иногда даже мелкие кости и волосы». Лечение также предложено в данном разделе: возможно использование прижигания или едких веществ, которые разъедают кожу вместе с оболочкой образования (при этом жидкостное содержимое вытекает, плотное содержимое отделяется вручную), или хирургическое лечение, при котором образование отделяется от здоровой ткани и удаляется вместе с капсулой – если это невозможно, следует прибегнуть к прижиганию [1][10].

Благодаря сочинениям Марка Витрувия (80–15 гг. до н. э.) и Плиния Старшего (23/24–79 гг.) можно предположить высокую распространенность зоба в Альпах. В своей работе под названием «Естественная история» (книга 9, глава 37) Плиний пишет: «Только люди и свиньи подвержены опухолям в горле; опухоли в основном вызваны плохим качеством потребляемой воды» [1][6][7].

Аретей из Каппадокии, живший в I–II веках, был выдающимся античным медиком, в своей практике придерживался учений Гиппократа. Именно он был первым, кто представил полное клиническое описание сахарного диабета, дав название заболеванию «διαβαίνω»=я прохожу сквозь [1][8]:

«… Диабет — это удивительное заболевание, представляющее собой расплавление плоти и конечностей в мочу. Эта болезнь не очень распространена. Жизнь пациентов коротка и болезненна; неутолимая жажда сочетается с чрезмерным питьем, которое, однако, несоизмеримо огромному количеству мочи. Ничто не может остановить от питья и произведения воды, но если на некоторое время они воздерживаются от питья, то у них пересыхает во рту и даже тело начинает сохнуть: внутренности словно обожжены, мучает тошнота, непреодолимая жажда; в результате в ближайшем будущем они истекают» [1].

В трактате «О голосе» («De voce»), авторство которого предписывается Галену, есть упоминание о щитовидной железе:

«…На шее есть две железы, в которых вырабатывается влага: в надгортаннике образуется густая и вязкая жидкость, необходимая для его увлажнения. Однако в них нет протоков, по которым жидкость может течь, как это происходит в двух железах языка…»

Длительное время теория Галена о том, что вещество, вырабатываемое щитовидной железой, необходимо для увлажнения гортани, сохранялась среди врачей, а отсутствие протока у щитовидной железы вызывало недоумение у анатомов.

Для лечения зоба древнеримский медик предлагал использовать жженую губку (spongia usta), сделанную из пузырчатого фукуса (fucus vesiculosus) [1].

Гален одним из первых высказал предположение о влиянии гипофиза на щитовидную железу. Он указал, что гипофиз окружен сосудистой сетью («rete mirabilis) и располагается в турецком седле, предположил, что через сосудистую сеть проходят нервные импульсы, которые и воздействуют на щитовидную железу [1][7][9].

## ЗАКЛЮЧЕНИЕ

История медицины — это неотъемлемая часть мировой культуры. Многочисленные работы древних авторов, посвященные медицине, на протяжении веков были основными врачебными пособиями. Некоторые достижения ученых того времени актуальны и сегодня. Так, выдающиеся мыслители Древнего мира не могли не затронуть такую отрасль медицины, как эндокринология. Уже тогда врачи не оставляли попыток найти причину появления того или иного заболевания, а главное, они пытались подобрать эффективный метод лечения. И сегодня, когда эндокринология является одной из самых быстроразвивающихся отраслей медицины, мы должны помнить о наследии Древнего мира, благодаря которому эндокринология и появилась.

## ДОПОЛНИТЕЛЬНАЯ ИНФОРМАЦИЯ

Источники финансирования. Работа выполнена по инициативе авторов без привлечения финансирования.

Конфликт интересов. Авторы декларируют отсутствие явных и потенциальных конфликтов интересов, связанных с написанием данной обзорной статьи и ее публикацией.

Участие авторов. Все авторы внесли значимый вклад в подготовку статьи, прочли и одобрили финальную версию.

* Тебе следует сделать: «shebeb»-смола 1/64 «hekt», смолы «auyt» … вместе [4].

** «И сказал Исав Иакову: дай мне поесть красного, красного этого, ибо я устал. От сего дано ему прозвание: Едом. Но Иаков сказал [Исаву]: продай мне теперь же свое первородство. Исав сказал: вот, я умираю, что мне в этом первородстве? Иаков сказал [ему]: поклянись мне теперь же. Он поклялся ему, и продал [Исав] первородство свое Иакову. И дал Иаков Исаву хлеба и кушанья из чечевицы; и он ел и пил, и встал и пошел; и пренебрег Исав первородство» (Бытие, гл. 25, ст. 29-34).

*** Первый вышел красный, весь, как кожа, косматый; и нарекли ему имя Исав (Бытие 25:22-25).

**** Babylonian Talmud (Tractate Shabbath, p. 53b).
